# Microstructure and Properties of SUS304 Stainless Steel Joints Brazed with Electrodeposited Ni-Cr-P Alloy Coatings

**DOI:** 10.3390/ma14154216

**Published:** 2021-07-28

**Authors:** Shubin Liu, Ikuo Shohji, Tatsuya Kobayashi, Katsuharu Osanai, Tetsuya Ando, Junichiro Hirohashi, Tsunehito Wake, Katsufumi Inoue, Hiroki Yamamoto

**Affiliations:** 1 Graduate School of Science and Technology, Gunma University, 1-5-1, Tenjin-cho, Kiryu 376-8515, Japan; shohji@gunma-u.ac.jp (I.S.); kobayashi.t@gunma-u.ac.jp (T.K.); 2 Graduate School, Muroran Institute of Technology, 27-1, Mizumoto-cho, Muroran 050-8585, Japan; 20042011@mmm.muroran-it.ac.jp (K.O.); ando@mmm.muroran-it.ac.jp (T.A.); 3 Waki Factory Inc., 6-760 Higashi-sayamagaoka, Tokorozawa 359-1106, Japan; j-hirohashi@waki-ss.co.jp (J.H.); t_wake@fc4.so-net.ne.jp (T.W.); k-inoue@waki-ss.co.jp (K.I.); 4 Kandori Industry Co., 57-1 Kamimutsuguri Kanegasaki, Kota-cho 444-0123, Japan; kamiroku@kandori.jp

**Keywords:** electrodeposited Ni-Cr-P coating, brazing filler metal, microstructure, shear strength

## Abstract

In this study, an amorphous Ni-13.4Cr-11.6P (mass%) alloy coating with a thickness of 30 μm was deposited on the surface of SUS304 stainless steel as a brazing filler metal to conduct brazing. The differential thermal analysis measurements indicate that the electrodeposited Ni-13.4Cr-11.6P alloy has a melting point of approximately 892 °C, which is almost consistent with that of the commercial BNi-7 filler metal. The microstructure, shear strength, and fracture mode of the brazed joint were investigated using an electron probe X-ray microanalyzer, a scanning electron microscope, an optical microscope, and a universal testing machine. The results showed that the brazed filler metal is filled between the SUS304 stainless steel plates without any flaws in the brazed seam. The P-containing phases, i.e., the Cr-P rich phase and the (Ni,Fe)_3_P phase, were formed in the brazed seam. The shear strength of the brazed joint obtained in this study is 59.0 MPa. The fracture occurs in the brazed filler zone, where the brittle P-containing phases are present. Galvanic current measurement results showed that the brazed Ni-13.4Cr-11.6P alloy coating has a better corrosion resistance than that of the brazed Ni-11P alloy coating, which can be attributed to the formation of a large amount of Ni-Fe solid solution and Cr-P rich phase in the top layer of the brazed Ni-13.4Cr-11.6P alloy coating.

## 1. Introduction

SUS304 stainless steel has been commonly used as a base material for heat exchangers, coolers, microchemical systems, and other high-temperature, high corrosion-resistant thin-wall pipe and plated parts owing to its excellent corrosion resistance and heat resistance. Diffusion bonding [1,2,3] and diffusion brazing (referring to brazing in this study) [4,5,6] are widely used for joining stainless steel. Compared to diffusion brazing, diffusion bonding can form a high-quality joint by overcoming the formation of brittle intermetallic compounds (IMCs), chemical segregation, and accumulation of residual stress at the joint interface, owing to its characteristics of solid-state diffusion. However, diffusion bonding is expensive because special and expensive equipment is required to meet the high heating temperatures and the controlled pressure in a vacuum or protective gas atmosphere [7]. Diffusion brazing commonly uses a thin interlayer (or filler metal) that has a lower melting point than the base materials and can melt below the bonding temperature. The interdiffusion of elements between the melted interlayer and the dissolved base materials near the interface of the joint causes isothermal solidification (or alloying), thus joining the base materials together. Since no relevant pressure is applied in the brazing process, the equipment is much simpler. In addition, in terms of economy, brazing is considered to be more suitable to meet mass-production requirements.

Ni-based brazing filler metals are commonly used for brazing stainless steel components of heat exchangers. In order to obtain a high-quality brazed joint, the choice of filler metals is very important. BNi-2, containing (2.75–3.5 mass%) B element, has been commonly used in brazing of stainless steel; however, when brazing thin-walled components, B tends to diffuse to the base material and form brittle Cr-B IMCs, causing grain boundary penetration embrittlement, and dissolution corrosion, thus reducing the reliability of brazed joints [8,9,10,11,12]. Compared with B, it is difficult for P to diffuse into the base material and generate IMCs in the bonding-affected zone due to its large atom radius and low diffusion rate [13]. Moreover, filler metals containing P have a lower melting point than those containing other melting point depressants (i.e., B, Si). In addition, Ni-based filler metal containing a certain amount of Cr (such as BNi-7) has been used to improve the heat resistance and corrosion resistance of the brazed joint. Many studies showed that the BNi-7 filler metal is suitable to braze stainless steel with a thin thickness owing to its excellent wettability and low melting point [14,15,16,17].

With the development of high-efficiency compact heat exchangers, for example, which can be used for solid oxide fuel cell (SOFC), the components have become thinner (0.2–0.3 mm) and more multi-layered. The heat exchanger is assembled by stacking dozens of such thin stainless-steel plates by brazing. Currently, there are two main difficulties in the miniaturization of heat exchangers. Firstly, due to the inherent hardness and brittleness of Ni-based filler metals, it is difficult for Ni-based filler metals to form a foil like copper brazing filler metals. It can only be used as a rod material of a few millimeters (mm) or a paste material of tens to hundreds of microns (μm). The use of these forms of filler metal makes the thickness of the brazed seam up to 100 μm, compared to the stainless steel plate of only 0.2–0.3 mm thickness, which greatly hinders the goal of miniaturization. In addition, the use of these forms of filler metal makes it difficult to supply the correct quality of the filler metal to the brazing area, which in turn leads to the degradation and decreases the reliability of brazed joints [18].

Secondly, Ni-based filler metals are extremely expensive. In such a stacked structure heat exchanger, the amount of filler metal used would be tremendous due to the large number of locations where the filler metal has to be applied, creating the problem of a large rise in cost. Therefore, it is especially important to develop low-cost techniques to address the challenges mentioned above. One well-known technique is electrodeposition, of which the main advantages are precise control of the chemical composition and thickness of the deposited alloys, as well as the capability to coat components with a complex geometry [19]. Considering production costs, electrodeposition is undoubtedly a good choice. According to our previous study [20], the electroplating method was used to deposit a Ni-P alloy coating with certain chemical composition and thickness on the surface of stainless steel, and the brazing of stainless steel with the electrodeposited Ni-P alloy coating was successfully realized. In order to meet the application of heat exchangers in the high temperature field (i.e., 500–1000 °C), a novel Ni-Cr-P plating bath was developed and a Ni-13.4Cr-11.6P (mass%) alloy coating with similar chemical composition to BNi-7 filler was successfully prepared on the surface of SUS304 plate [21].

In addition, it has been found that the stainless-steel heat exchangers brazed with Ni-based filler metals may lead to water leakage after long-term usage, which can be attributed to the corrosion caused by Cl^−^ ions in the aqueous solution.

Therefore, in this study, electrodeposited Ni-13.4Cr-11.6P alloy coating was used as the brazing filler metal to conduct the brazing. The microstructure, shear strength, and fracture mode of the brazed joint were investigated. In addition, the corrosion behaviors of the brazed Ni-13.4Cr-11.6P alloy coating were investigated using galvanic current measurements.

## 2. Materials and Methods

### 2.1. Materials

A SUS304 stainless steel plate with a thickness of 1.5 mm was used as the base metal to conduct the brazing tests. The chemical composition of SUS304 stainless steel is given in Table 1. A novel developed plating solution (shown in Table 2) was used to deposit the Ni-Cr-P alloy coating [21]. The chemical composition of the electrodeposited Ni-Cr-P alloy coating analyzed by X-ray fluorescence (XRF, Shimadzu XRF-1700, Kyoto, Japan) is also given in Table 1. The electrodeposited Ni-Cr-P alloy coatings have a thickness of approximately 30 μm, which was determined by measuring the thickness difference before and after electroplating on the surface of SUS304 plate with a micrometer.

**Figure 1:**
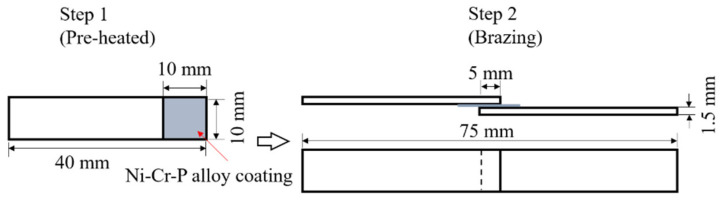
Geometry and dimensions of specimen for brazing.

Before plating, the SUS304 plate was mechanically polished with #1000 and #2400 emery papers, degreased with acetone, etched in 10% HCl solution for 2.5 min to remove the oxide film on the surface of stainless steel, rinsed with pure water, and dried with a dryer. After plating, the SUS304 plate electrodeposited with Ni-Cr-P alloy coating was cut in a size shown in Figure 1. The crystallographic structure of the electrodeposited Ni-13.4Cr-11.6P alloy coating was investigated using X-ray diffraction (XRD, Rigaku RINT 2200 VF, Tokyo, Japan) analysis at 40 kV and 20 mA with Cu kα radiation at a scan rate of 0.02 °/s in the 2*θ* range of 20–80°. For XRD analysis, the alloy coating was electrodeposited on a Cu plate to avoid interference from Cr and Ni in SUS304.

The melting point of the electrodeposited Ni-13.4Cr-11.6P alloy coating was measured using a differential thermal analysis (DTA; TG/DTA7300, HITACHI, Tokyo, Japan) system in a high pure Ar atmosphere. The flow quantity of Ar gas was 200 mL/min. The weight of the specimen used for measurement was 1.58 mg. The specimen was heated from 20 °C to 1150 °C with a heating rate of 10 °C/min.

### 2.2. Brazing Test

The brazing of SUS304 plates with the electrodeposited Ni-Cr-P alloy coating was performed using a hydrogen reduction furnace with a dew point of −50 °C. The brazing temperature was set to 1020 °C and the holding time at the brazing temperature was 15 min. The heating time from room temperature to the brazing temperature and the cooling time from the brazing time to room temperature were approximately 30 and 45 min. To prevent the sensitization of SUS304, the cooling time from 800 °C to 600 °C was controlled less than 4 min. The flow quantity of hydrogen gas was 7.5 m^3^/h. In order to remove the O and C impurities in the electrodeposited alloy coating, the Ni-Cr-P alloy coating on the SUS304 plate was pre-heated using the hydrogen reduction furnace in the temperature profile same as brazing. Afterward, assembled the pre-heated SUS304 plates as shown in Figure 1, and secured them with a thin steel wire. Subsequently, the assembled specimen was brazed under the same brazing conditions.

### 2.3. Shear Test

A shear test was performed at room temperature with a universal testing machine (5567, Instron Japan Co., Ltd., Kanagawa, Japan). The cross-head speed was set to 10 mm/min. The geometry and dimensions of the specimen used for the shear test are the same, as shown in Figure 1 (Step 2). The fracture mode observation of the brazed joint was performed using a scanning electron microscope (SEM; HITACHI S-4300SE/N, Tokyo, Japan) and an optical microscope (OM; VK-X150, KEYENCE, Osaka, Japan).

### 2.4. Microstructure Observation

The brazed joints used for microstructure analysis were prepared according to the standard metallographic techniques. Specimens for cross-sectional investigation were cut with a fine cutter, embedded in epoxy resin, and abraded with #320, #500, #800, #1200, #2400, and #4000 emery papers, and subsequently polished with a 1.0 µm Al_2_O_3_ suspension. The microstructure analysis of the brazed joint was conducted with an electron probe X-ray microanalyzer (EPMA; EPMA-1610, Shimadzu, Kyoto, Japan) at an acceleration voltage of 15 kV.

### 2.5. Electrochemical Analysis

**Figure 2:**
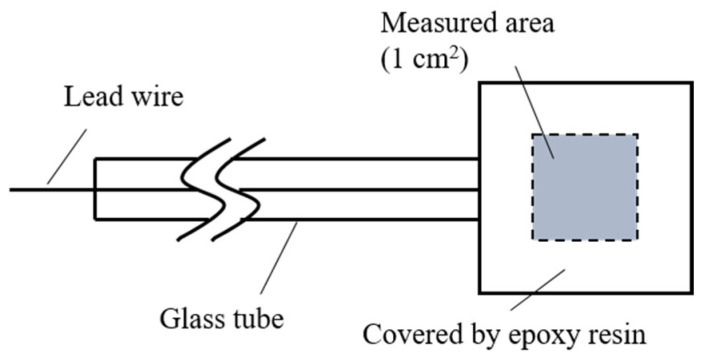
Schematic of specimen used for spontaneous potential and galvanic current measurements.

A SUS304 plate electrodeposited with a 30 μm thickness Ni-13.4Cr-11.6P alloy coating was used for spontaneous potential and galvanic current measurements. The alloy coating was heat-treated using the same brazing conditions mentioned in Section 2.2. Figure 2 shows the schematic of the specimen used for the spontaneous potential and galvanic current measurements. The specimen was coated by epoxy resin with an exposed area of 1 cm^2^. Before measurements, the exposed area was polished with a #1000 emery paper and cleaned with pure water.

The spontaneous potential and galvanic current measurements were conducted in a 0.06 M/L NaCl aqueous solution. For spontaneous potential measurement, a saturated silver chloride electrode (Ag/AgCl) was used as the reference electrode. The specimen and the reference electrode were set as positive and negative electrodes, respectively. The volume of the test solution was 200 mL and the distance between the two electrodes was set to 40 mm. The measurement was initially performed at 25 °C for 24 h, then at 80 °C for 72 h, and subsequently restored the solution temperature to 25 °C for 24 h. For galvanic current measurement, the SUS304 plate and the specimen were set as positive and negative electrodes of a zero-shunt ammeter, respectively. The measurement was performed under the same temperature profile as for spontaneous potential measurement. The microstructure observation before and after the galvanic current measurement was conducted with the EPMA.

## 3. Results and Discussion

### 3.1. Properties of Electrodeposited Ni-13.4Cr-11.6P Alloy Coating

**Figure 3:**
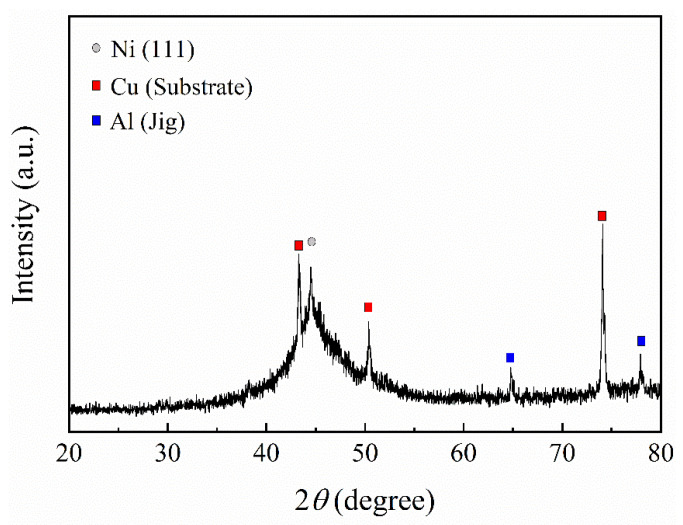
XRD diffraction pattern of electrodeposited Ni-13.4Cr-11.6P alloy coating.

The XRD diffraction pattern of the electrodeposited Ni-13.4Cr-11.6P is presented in Figure 3. The diffractogram presents a broad peak around 2*θ* of 44.8° (characteristic of Ni (111) peak). This means that the electrodeposited Ni-13.4Cr-11.6P alloy coating has an amorphous structure. The formation of amorphous structure can be ascribed to the incorporation of phosphorus in the deposit [22,23,24]. In addition, the sharp peaks in the XRD diffraction pattern originated from the Cu substrate and the Al jig.

**Figure 4:**
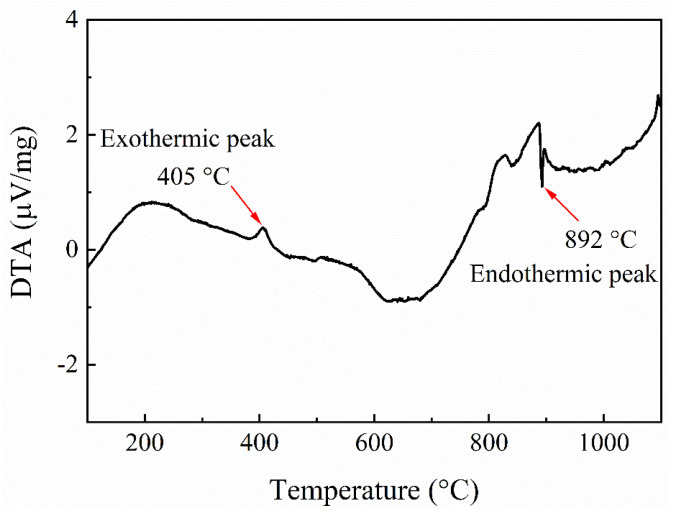
DTA curve of electrodeposited Ni-13.4Cr-11.6P alloy coating.

The melting point of the electrodeposited Ni-13.4Cr-11.6P alloy coating was measured by DTA and the measured curve is shown in Figure 4. The weak exothermic peak at 405 °C indicated that the alloy coating crystallized at this temperature [25]. The sharp endothermic peak appeared at 892 °C, indicating that the melting point of the electrodeposited Ni-13.4Cr-11.6P alloy coating is 892 °C. This measured value is almost consistent with the melting point of BNi-7 (JIS Z 3265) filler metal.

### 3.2. Microstructure Analysis of Brazed Joint

**Figure 5:**
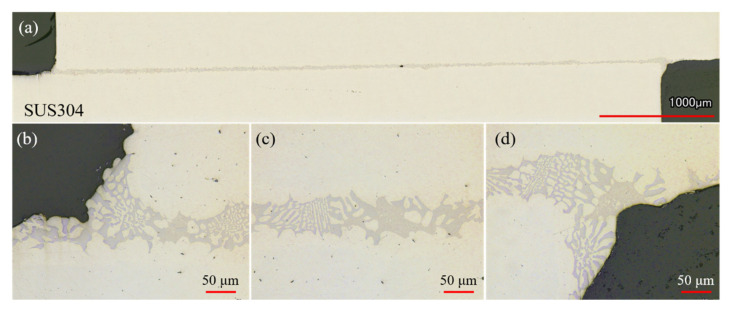
OM images of cross section of joint brazed with electrodeposited Ni-13.4Cr-11.6P alloy coating. (**a**) is overall view of brazed joint, (**b**–**d**) are partically magnified images of (**a**).

Figure 5 shows the OM images of the joint brazed with the electrodeposited Ni-13.4Cr-11.6P alloy coating. It can be seen that the filler metal shows good bondability and no defects such as voids were formed in the brazed seam, indicating that the brazing of SUS304 plates with the electrodeposited Ni-13.4Cr-11.6P alloy coating was successfully realized. In addition, Figure 5b,d show that an incomplete fillet is formed at both ends of the brazed joint, which can be associated with the insufficient volume of filler metal during the brazing process.

**Figure 6:**
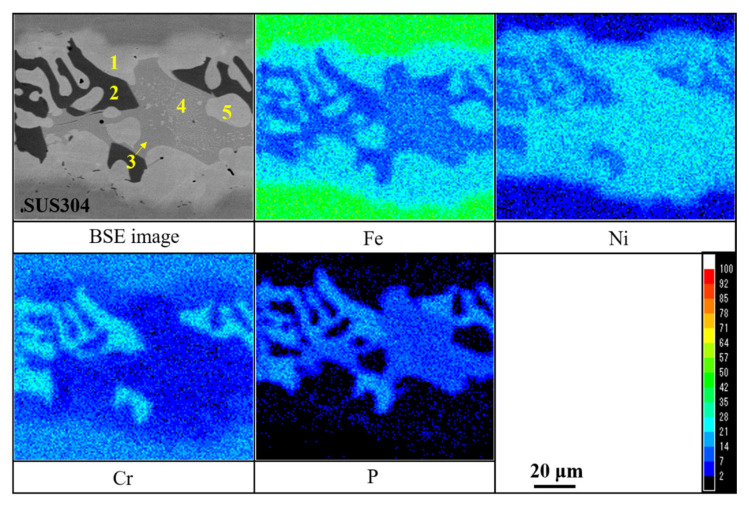
Mapping analysis results for cross section of joint brazed with electrodeposited Ni-13.4Cr-11.6P alloy coating.

Figure 6 shows the mapping analysis results for the cross section of joint brazed with the electrodeposited Ni-13.4Cr-11.6P alloy coating. As seen in the backscatter electron (BSE) image, there are five phases formed in the brazed seam. The bright gray phase, marked as point 1, existed at the brazed interfaces of the SUS304 plates mainly contains Fe and Ni elements. The dark gray phase, marked as point 2, distributed among the brazed seam mainly contains Cr and P elements. The light gray phase, marked as point 3 and 4, adjacent to the dark gray phase seems to be comprised of two different phases, the light gray phase and a large number of micro-particles. The last one (marked as point 5) is the island-like phase dispersed in the brazed seam mainly contains Fe and Ni elements.

**Figure 7:**
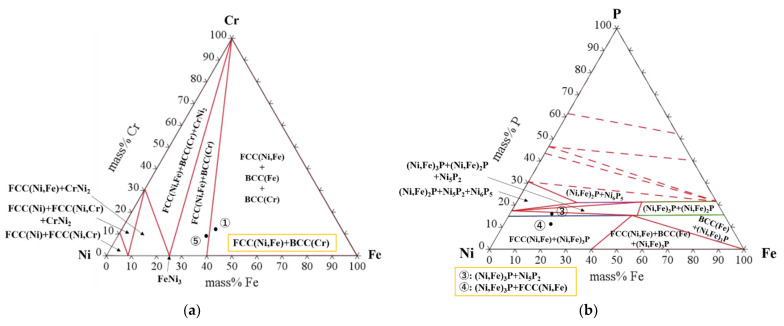
Phase diagrams of ternary Ni-Fe-Cr alloys (**a**) and Ni-Fe-P alloys (**b**) calculated by Therm-Clac 2021 (25 °C).

Quantitative analysis was performed to identify these phases formed in the brazed seam. The chemical compositions of these phases are shown in Table 3. It can be seen that Fe and Ni are main composition in the bright gray phase (point 1). In addition, a small amount of Cr and tiny amounts of P are contained in this phase. According to the isothermal section of the Ni-Fe-Cr phase diagram shown in Figure 7a (calculated by Thermo-Clac 2021 (using SSOL6: SGTE Alloy Solutions Database v6.0)), the bright gray phase was identified as Ni-Fe solid solution, in which a small amount of Cr phase was contained. The dark gray phase (point 2) contains Ni, Cr, Fe and P, which makes the phase more complicated. However, because the content of Cr and P is high, thus the dark gray phase can be inferred to be Cr-P rich phase. As shown in Table 3, the light gray phases marked as point 3 and 4 have a slight difference in the chemical composition, which can be ascribed to the interference of the micro-particles. These micro-particles are too small to analyze by the EPMA. However, from Figure 6, these micro-particles seem to be identical with the bright gray phase (point 1). According to the isothermal section of the Ni-Fe-P ternary phase diagram (shown in Figure 7b), the phase represented by point 3 was identified as (Ni,Fe)_3_P phase containing a small amount of Ni_5_P_2_ compounds, while the phase represented by point 4 was identified as (Ni,Fe)_3_P phase containing a small amount of Ni-Fe solid solution. Therefore, it can be inferred that the micro-particles are Ni-Fe solid solution. Since the two phases are mainly composed of (Ni,Fe)_3_P phase, it can be inferred that the two phases are the same. The island-like phase (point 5) has a chemical composition basically the same as the bright gray phase (point 1). Therefore, the phase of point 5 can be identified as Ni-Fe solid solution.

### 3.3. Shear Strength

Shear test was performed to investigate the mechanical properties of the brazed joints. The shear strength of the brazed joints is shown in Table 4. In addition, the shear strength of joints brazed with the electrodeposited Ni-11P alloy coating obtained in our previous study [20] is also presented in Table 4 for comparison. The shear strength of joint brazed with the electrodeposited Ni-13.4Cr-11.6P alloy coating is 59.0 MPa, which is higher than that of joint brazed with the electrodeposited Ni-11P alloy coating.

**Figure 8:**
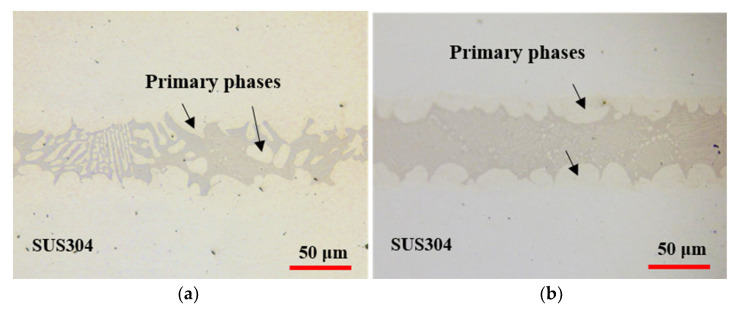
OM images of cross sections of joints brazed with electrodeposited Ni-13.4Cr-11.6P alloy coating (**a**) and electrodeposited Ni-11P alloy coating (**b**).

P mainly distributed in the filler metal zone to form the brittle P-containing compounds owing to its large atomic radius and slow diffusion rate [13,26,27]. As shown in Figure 8a, a large number of island-like phases are formed in the brazed seam, breaking the continuous brittle phase (compared with Figure 8b) into small fragments, which in turn improves the strength of the brazed joint.

The shear strength obtained in this study seems to be lower than the reported ones [14,15,16,17]. However, it is well known that there are many parameters that affect the shear strength of the brazed joint, such as the brazing temperature, the holding time at brazing temperature and the gap width of the joint. Zhang et al. [14] reported that the shear strength of the brazed joint increased from 60 MPa to 160 MPa with an increase in the brazing temperature. Li et al. [15] studied the effect of gap width and holding time on the mechanical properties of the brazed joint. They found that proper extension of the holding time and proper reduction of the gap width of the brazing joint can effectively promote the diffusion of elements between the filler metal and the base metal, which facilitates the formation of primary phase in the brazed seam, interrupting the continuity of the brittle P-containing compounds and improving the strength of the brazed joint.

In addition, the insufficient fillet formation at both ends of the brazed joint is also a major reason cause of the low shear strength. Although the shear strength obtained in this study is low, the brazed joint shows an excellent quality without any defects. From this perspective, the brazing of SUS304 plates using the electrodeposited Ni-13.4Cr-11.6P alloy coating as filler metal was successfully realized. A further study will be conducted to obtain a deeper understanding on the effect of brazing conditions on the shear strength of the brazed joint.

### 3.4. Fracture Mode of Brazed Joint

**Figure 9:**
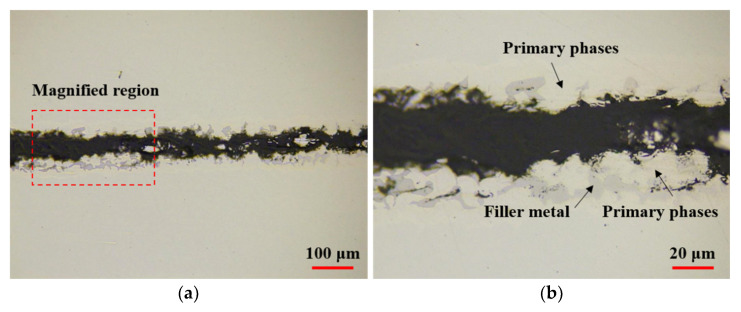
OM images of cross section of fractured joint brazed with electrodeposited Ni-13.4Cr-11.6P alloy coating. (**b**) is magnified region in (**a**).

Figure 9 shows the OM images of cross section of the fractured brazed joint by shear test. As shown in Figure 9b, the cracks arose at the interface between the brittle compound (i.e., P-containing phase) and the primary phase, and the cracks extended along this interface, eventually leading to breakage.

### 3.5. Corrosion Behaviors

**Figure 10:**
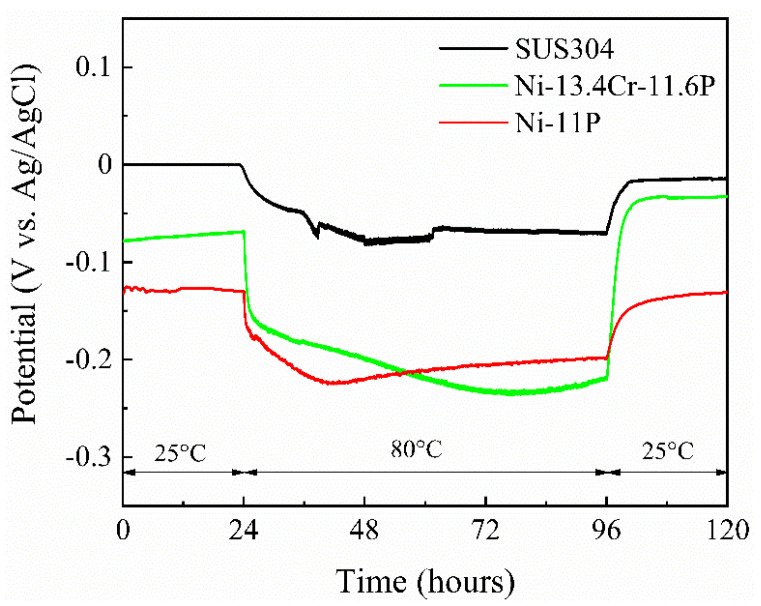
Spontaneous potentials of SUS304, brazed Ni-11P alloy coating, and brazed Ni-13.4Cr-11.6P alloy coating in 0.06 M/L NaCl aqueous solution at different temperatures.

Figure 10 shows the spontaneous potentials of SUS304, brazed Ni-11P alloy coating, and brazed Ni-13.4Cr-11.6P alloy coating measured in a 0.06 M/L NaCl solution. The spontaneous potentials of the brazed alloy coatings are more negative than that of SUS304 at different temperatures, indicating that the brazed alloy coatings are pone to become the anode materials in corrosion process, i.e., they are easy to be corroded. It is worth noting that the potentials of the three specimens shifted negatively as the temperature increased to 80 °C. This phenomenon can be associated with the fact that the high temperature accelerates the destructive effect of Cl^−^ ions on the oxide film on the surfaces of the specimens.

**Figure 11:**
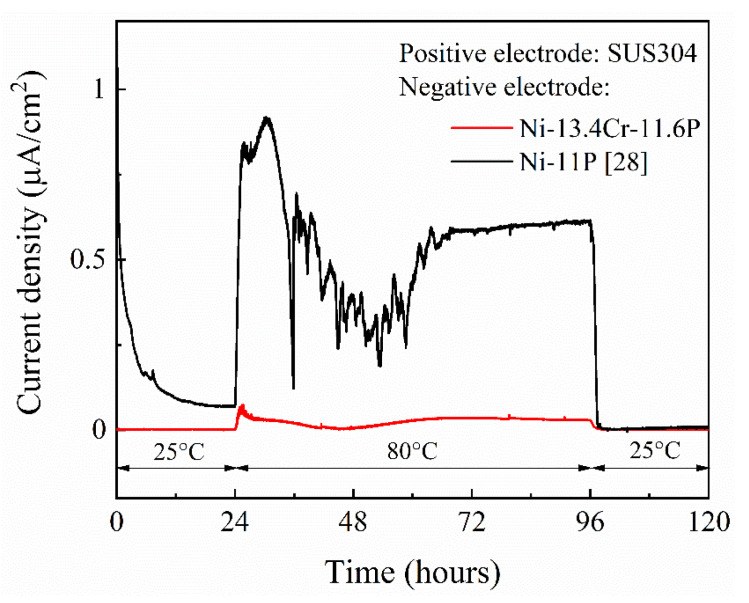
Current density-time curve of galvanic couples of SUS304/brazed Ni-11P alloy coating and SUS304/brazed Ni-13.4Cr-11.6P alloy coating in 0.06 M/L aqueous solution at different temperatures.

Figure 11 shows the current density-time curve of galvanic couples of SUS304/brazed Ni-11P alloy coating [28] and SUS304/brazed Ni-13.4Cr-11.6P alloy coating measured in a 0.06 M/L NaCl solution. At a temperature of 25 °C, current scarcely flowed in both of the two galvanic couples during the first 24 h of immersion. With the temperature increased to 80 °C, a current density of up to 1.0 μA/cm^2^ flowed in the SUS304/brazed Ni-11P alloy coating galvanic couple, indicating that the brazed Ni-11P alloy coating was corroded. At the same temperature, only a current density of up to 0.07 μA/cm^2^ flowed in the SUS304/brazed Ni-13.4Cr-11.6P alloy coating galvanic couple, suggesting that the brazed Ni-13.4Cr-11.6P alloy coating has a better corrosion resistance than that of the brazed Ni-11P alloy coating. The current flow in the galvanic couples can be attributed to the increasing in potential difference between SUS304 and the brazed alloy coatings at 80 °C (as shown in Figure 10), which acts as a driving force to promote the corrosion process [29]. When the temperature restored to 25 °C, the current density of both galvanic couples dropped to approximately 0 μA/cm^2^.

**Figure 12:**
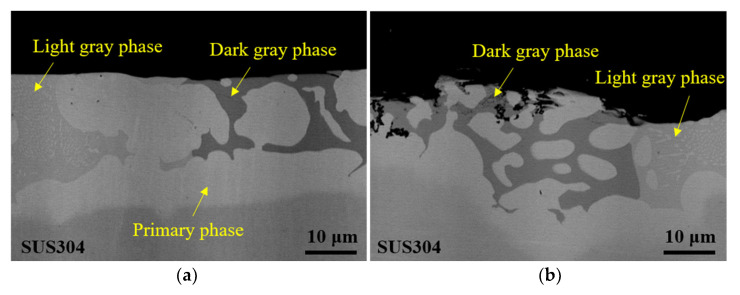
BSE images of cross sections of brazed Ni-13.4Cr-11.6P alloy coating. (**a**) Before galvanic current measurement; (**b**) after galvanic current measurement.

Figure 12 shows the BSE images of cross sections of the brazed Ni-13.4Cr-11.6P alloy coating before and after the galvanic current measurement. Before the measurement, the surface of the specimen is flat, while after the measurement, its surface becomes uneven. As seen in Figure 12b, the P containing phases, i.e., Cr-P rich phase and (Ni,Fe)_3_P phase, were corroded after the measurement. Masanori [30] reported that the increase of P content accelerating the corrosion process can be attributed to the formation of phosphide, which decreased the cathode overvoltage. From Figure 12b, it seems that the corrosion of the light gray phase (i.e., (Ni,Fe)_3_P phase) is more severe than that of the dark gray phase (i.e., Cr-P rich phase). Although the P content in the light gray phase is lower than that in the dark gray phase, the Cr content in the dark gray phase is much higher (seen in Table 3). Due to the high corrosion resistance of Cr, it is extremely easy to form an oxide film to inhibit the corrosion process.

**Figure 13:**
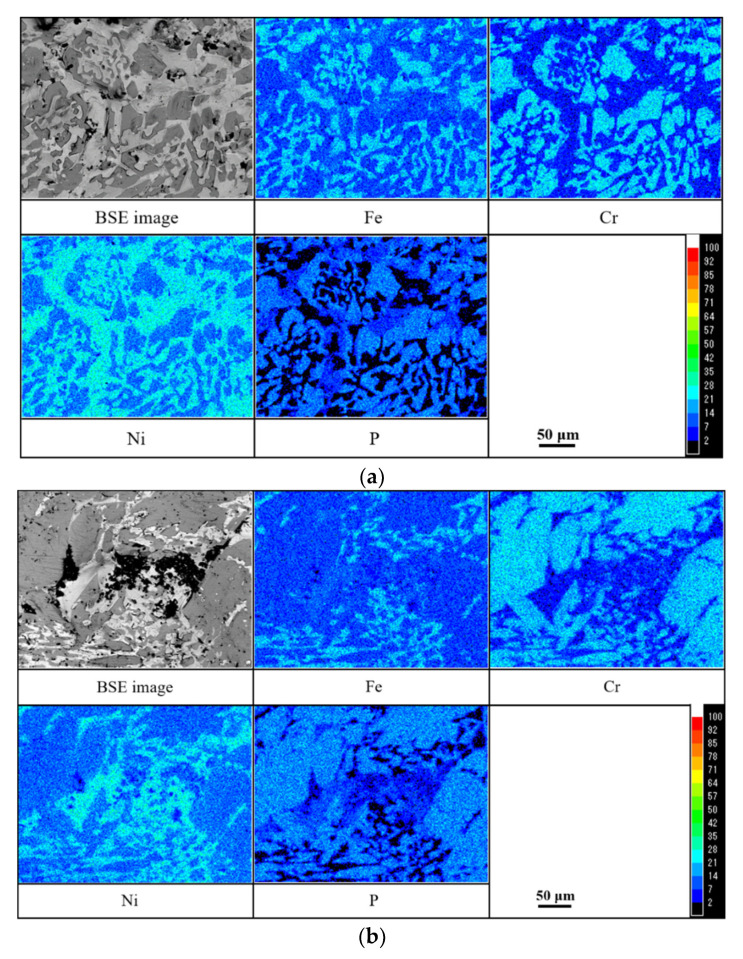
Mapping analysis for surface of brazed Ni-13.4Cr-11.6P alloy coating. Before galvanic current measurement (**a**) and after galvanic current measurement (**b**).

Mapping analysis for the surface of the brazed Ni-13.4Cr-11.6P alloy coating before and after the galvanic current measurement are shown in Figure 13. As shown in Figure 13a, a large number of Ni-Fe solid solution and Cr-P rich phase were observed in the top layer of the brazed alloy coating. These phases play a decisive role in inhibiting the corrosion process. After the measurement, the Cr-P rich phase and the Ni-Fe solid solution were the main structures that remained on the surface of the corroded alloy coating (seen in Figure 13b), which also confirmed the above discussion.

## 4. Conclusions

This study presented a novel brazing technology for SUS304 stainless steel with an electrodeposited Ni-13.4Cr-11.6P alloy coating as filler metal. DTA measurement result showed that the melting point of the electrodeposited alloy coating was approximately 892 °C. An excellent joint with shear strength of 59.0 MPa was obtained by a step-wise brazing method at 1020 °C for 30 min. The brazed seam is mainly composed of Cr-P rich phase, (Ni,Fe)_3_P phase, Fe-Ni solid solution, and island-like Ni-Fe solid solution particles. Ni-Fe solid solution existed at the brazed interfaces of the SUS304 plates. P mainly concentrated in the brazed filler zone in the form of Cr-P rich phase and (Ni,Fe)_3_P phase. The island-like Ni-Fe solid solution particles dispersed in the P-containing phase. Fracture mode observation showed that the cracks extended along the interface between the brittle P-containing phase and the primary phase, resulting in fracture.

In addition, the corrosion behaviors of the brazed Ni-13.4Cr-11.6P alloy coating was investigated by galvanic current measurement. A maximal current density of 0.07 μA/cm^2^ flowed in the SUS304/brazed Ni-13.4Cr-11.6P alloy coating galvanic couple, which is lower than that of 1.0 μA/cm^2^ in the SUS304/brazed Ni-11P alloy coating galvanic couple, indicating that the brazed Ni-13.4Cr-11.6P alloy coating has a better corrosion resistance. The enhanced corrosion resistance can be attributed to the formation of a large amount of Ni-Fe solution and Cr-P rich phase in the brazed Ni-13.4Cr-11.6P alloy coating.

## Figures and Tables

**Table 1:** Chemical composition of SUS304 and electrodeposited Ni-Cr-P alloy coating (mass%).

Symbol	Fe	Ni	Cr	S	Si	P	Mn	C
SUS304	Bal.	8.00~10.50	18.00~20.00	≤0.030	≤1.00	≤0.045	≤2.00	≤0.08
-	**Ni**	**Cr**	**P**	**O**	**C**	-	-	-
Ni-Cr-P	Bal.	13.4	11.6	8.5	3.4	-	-	-

**Table 2:** Bath formulation and deposition conditions for electrodeposition of Ni-Cr-P alloy coating.

Composition	Concentration (M)	Deposition Conditions
CrCl_3_·6H_2_O (Chromium Chloride)	0.4	Current density (*j*): 15 A/dm^2^ pH: 1.8 Bath temperature: 30 °C Total charge: 1215 C/cm^2^ Anode: Platinum plated titanium mesh Cathode: SUS304 plate (with a plating area of 5 cm^2^)
NiCl_2_·6H_2_O (Nickel Chloride)	0.25	
NaHPO_2_·H_2_O (Sodium Hypophosphite)	0.14	
H_2_NCH_2_COOH (Glycine)	0.5	
NH_4_Cl (Amonium Chloride)	0.5	
NaCl (Sodium Chloride)	0.5	
C_6_H_5_Na_3_O_7_ (Sodium Citrate)	0.2	
NaBr (Sodium Bromide)	0.145	
H_3_BO_3_ (Boric Acid)	0.5	
C_7_H_4_NO_3_SNa·2H_2_O (Saccharin Sodium)	0.6 g/L	
SDS (Sodium Dodecyl Sulfate)	0.1 g/L	

**Table 3:** Chemical compositions of each phase shown in BSE image in Figure 6.

Point	Chemical Compositions (Mass%)			
	Ni	Cr	Fe	P
1	50.77	11.42	37.01	0.80
2	28.94	34.98	14.43	21.65
3	58.14	7.53	18.37	15.96
4	59.37	6.76	22.59	11.28
5	53.17	9.22	36.79	0.82

**Table 4:** Shear strength of joints brazed with electrodeposited Ni-13.4Cr-11.6P alloy coating and electrodeposited Ni-11P alloy coating [20].

Filler Metal	Sample No.	Shear Strength (MPa)	Ave. (MPa)
Ni-13.4Cr-11.6P	A-1	57.0	59.0
	A-2	60.0	
	A-3	59.4	
Ni-11P	B-1	54.3	47.3
	B-2	44.7	
	B-3	42.8	
	B-4	47.3	

## Data Availability

The data presented in this study are available on request from the corresponding author.
