# Correction: Liu et al. Microstructure and Properties of SUS304 Stainless Steel Joints Brazed with Electrodeposited Ni-Cr-P Alloy Coatings. *Materials* 2021, *14*, 4216

**DOI:** 10.3390/ma14195499

**Published:** 2021-09-23

**Authors:** Shubin Liu, Ikuo Shohji, Tatsuya Kobayashi, Katsuharu Osanai, Tetsuya Ando, Junichiro Hirohashi, Tsunehito Wake, Katsufumi Inoue, Hiroki Yamamoto

**Affiliations:** 1 Graduate School of Science and Technology, Gunma University, 1-5-1, Tenjin-cho, Kiryu 376-8515, Japan; shohji@gunma-u.ac.jp (I.S.); kobayashi.t@gunma-u.ac.jp (T.K.); 2 Graduate School, Muroran Institute of Technology, 27-1, Mizumoto-cho, Muroran 050-8585, Japan; 20042011@mmm.muroran-it.ac.jp (K.O.); ando@mmm.muroran-it.ac.jp (T.A.); 3 Waki Factory Inc., 6-760 Higashi-sayamagaoka, Tokorozawa 359-1106, Japan; j-hirohashi@waki-ss.co.jp (J.H.); t_wake@fc4.so-net.ne.jp (T.W.); k-inoue@waki-ss.co.jp (K.I.); 4 Kandori Industry Co., 57-1 Kamimutsuguri Kanegasaki, Kota-cho 444-0123, Japan; kamiroku@kandori.jp

Error in Figure/Table

In the original article [1], there was a mistake in “*Figure 10. Spontaneous potentials of SUS304, brazed Ni-11P alloy coating, and brazed Ni-13.4Cr-11.6P alloy coating in 0.06 M/L NaCl aqueous solution at different temperatures. Figure 11. Current density-time curve of galvanic couples of SUS304/brazed Ni-11P alloy coating and SUS304/brazed Ni-13.4Cr-11.6P alloy coating in 0.06 M/L NaCl aqueous solution at different temperatures.*” as published. “The 80 °C marked in Figure 10 and Figure 11 should be changed to 60 °C. Add NaCl to the caption of Figure 11.” The corrected “*Figure 10. Spontaneous potentials of SUS304, brazed Ni-11P alloy coating, and brazed Ni-13.4Cr-11.6P alloy coating in 0.06 M/L NaCl aqueous solution at different temperatures. Figure 11. Current density-time curve of galvanic couples of SUS304/brazed Ni-11P alloy coating and SUS304/brazed Ni-13.4Cr-11.6P alloy coating in 0.06 M/L NaCl aqueous solution at different temperatures.*” appears below. The authors apologize for any inconvenience caused and state that the scientific conclusions are unaffected. The original article has been updated.

Text Correction

There was an error in the original article. “The 80 °C in Section 2.5. Electrochemical Analysis and Section 3.5. Corrosion Behaviors should be changed to 60 °C”.

A correction has been made to “*Section 2. Materials and Methods and Section 3. Results and Discussion”, “Section 2.5. Electrochemical Analysis and Section 3.5. Corrosion Behaviors”, “Pages 5 of 14, 9 of 14, and 10 of 14*”:

“The spontaneous potential and galvanic current measurements were conducted in a 0.06 M/L NaCl aqueous solution. For spontaneous potential measurement, a saturated silver chloride electrode (Ag/AgCl) was used as the reference electrode. The specimen and the reference electrode were set as positive and negative electrodes, respectively. The volume of the test solution was 200 mL and the distance between the two electrodes was set to 40 mm. The measurement was initially performed at 25 °C for 24 h, then at 60 °C for 72 h, and the solution temperature was subsequently restored to 25 °C for 24 h. For galvanic current measurement, the SUS304 plate and the specimen were set as positive and negative electrodes of a zero-shunt ammeter, respectively. The measurement was performed under the same temperature profile as for spontaneous potential measurement. The microstructure observation before and after the galvanic current measurement was conducted with the EPMA. 

Figure 10 shows the spontaneous potentials of SUS304, brazed Ni-11P alloy coating, and brazed Ni-13.4Cr-11.6P alloy coating measured in a 0.06 M/L NaCl solution. The spontaneous potentials of the brazed alloy coatings are more negative than that of SUS304 at different temperatures, indicating that the brazed alloy coatings are prone to become anode materials in the corrosion process, i.e., they are easily corroded. It is worth noting that the potentials of the three specimens shifted negatively as the temperature increased to 60 °C. This phenomenon may be associated with the fact that the high temperature accelerates the destructive effect of Cl^−^ ions on the oxide film on the surfaces of the specimens. 

Figure 11 shows the current density-time curve of galvanic couples of SUS304/brazed Ni-11P alloy coating [28] and SUS304/brazed Ni-13.4Cr-11.6P alloy coating measured in a 0.06 M/L NaCl solution. At a temperature of 25 °C, current scarcely flowed in both of the galvanic couples during the first 24 h of immersion. With the temperature increased to 60 °C, a current density of up to 1.0 µA/cm^2^ flowed in the SUS304/brazed Ni-11P alloy coating galvanic couple, indicating that the brazed Ni-11P alloy coating was corroded. At the same temperature, only a current density of up to 0.07 µA/cm^2^ flowed in the SUS304/brazed Ni-13.4Cr-11.6P alloy coating galvanic couple, suggesting that the brazed Ni-13.4Cr-11.6P alloy coating has a better corrosion resistance than that of the brazed Ni-11P alloy coating. The current flow in the galvanic couples can be attributed to the increase in potential difference between SUS304 and the brazed alloy coatings at 60 °C (as shown in Figure 10), which acts as a driving force to promote the corrosion process [29]. When the temperature was restored to 25 °C, the current density of both galvanic couples dropped to approximately 0 µA/cm^2^.

The authors apologize for any inconvenience caused and state that the scientific conclusions are unaffected. The original article has been updated.

## Figures and Tables

**Figure 10:**
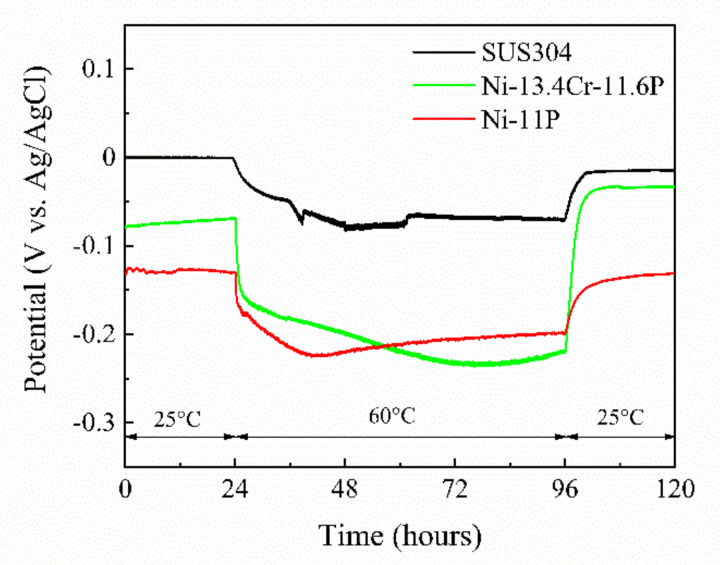
Spontaneous potentials of SUS304, brazed Ni-11P alloy coating, and brazed Ni-13.4Cr-11.6P alloy coating in 0.06 M/L NaCl aqueous solution at different temperatures.

**Figure 11:**
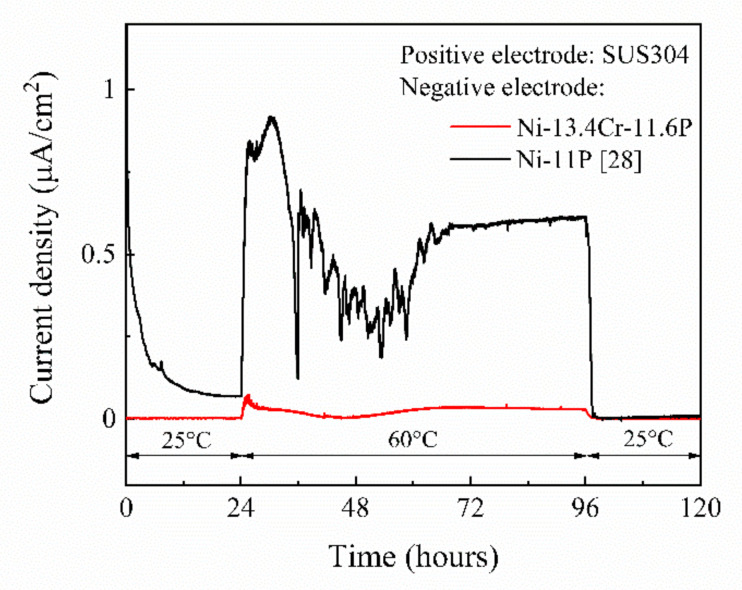
Current density-time curve of galvanic couples of SUS304/brazed Ni-11P alloy coating and SUS304/brazed Ni-13.4Cr-11.6P alloy coating in 0.06 M/L NaCl aqueous solution at different temperatures.
